# A facet joint landmark-guided approach for multi-segmental DRG injection with limited bone removal in mice

**DOI:** 10.3389/fneur.2026.1956927

**Published:** 2026-10-15

**Authors:** Ruohan Yan, Sike Sun, Zipeng Zhou, Lei Ma, Junhao Deng, Jinhui Zhang

**Affiliations:** 1 State Key Laboratory of Natural Medicines, China Pharmaceutical University, Nanjing, China; 2 School of Life Science and Technology, China Pharmaceutical University, Nanjing, China; 3 Department of Orthopedics, Senior Department of Orthopedics, Chinese PLA Hospital, Beijing, China; 4 School of Life Science, Tsinghua University, Beijing, China; 5 Department of Orthopaedics, The 909th Hospital, School of Medicine, Xiamen University, Zhangzhou, China

**Keywords:** dorsal root ganglion, facet joint landmark, *in vivo* DRG imaging, neurogenic pain, viral vector delivery

## Abstract

**Background:**

Targeted gene delivery to the dorsal root ganglion (DRG) has been developed as a valuable approach for pain research. Nevertheless, conventional DRG microinjection approaches suffer from notable drawbacks: vertebral bone resection and facet joint destruction induce spinal instability, extensive tissue adhesion impedes repeated surgery, and multi-segment targeting remains challenging.

**Methods:**

To establish a multi-segmental DRG injection workflow, we developed a vertebral counting scheme based on mouse anatomical dissection. To guide our selection of target segments for viral delivery, Thy1-YFP-H transgenic mice was used to trace segmental DRG innervation patterns with good resolution. Anatomical dissection was then performed to examine the precise spatial relationship between each target DRG and its neighboring facet joints. With this anatomical roadmap, together with a custom designed temporary spinal stabilizer, we established a standardized injection procedure applicable to user-specified levels. Post-operatively, animal behavior was serially assessed, and finally *in vivo* GCaMP calcium imaging was conducted to verify functional viral transduction.

**Results:**

Vertebral counting based on the V-shaped white lines on the spinous processes enabled reliable identification of lumbar segmentation. YFP tracing clarified distinct segmental innervation patterns: L1-L2 fibers do not contribute to the sciatic nerve; L3-L5 collectively form the sciatic nerve; L6 fibers, plus minor L5 branches, constitute the pudendal nerve. Additional anatomical dissection confirmed that drilling at the base of the inferior articular process of the cranial vertebra provided a direct access route to the L3-L6 DRGs. Serial behavioral monitoring post-injection revealed transient functional disturbances, most of which resolved by postoperative day 21. Finally, concurrent jGCaMP8s imaging confirmed successful functional transduction: air-puff stimulation elicited robust calcium responses in transduced DRG neurons, whereas no such signals were detected in wild-type controls.

**Conclusion:**

We have established a reproducible viral delivery method that minimizes bone resection and avoids gross facet joint destruction through laminar drilling, while enabling multi-segmental injection into mouse L3-L6 DRGs. The combination of longitudinal behavioral monitoring and stimulus-evoked calcium imaging supported the surgical feasibility and functional transduction of this approach. This method thus provides an experimental toolkit that should be useful for dissecting somatic-visceral pain crosstalk through multi-segmental DRG genetic manipulation.

## Introduction

The dorsal root ganglion (DRG) contains somata of primary afferent neurons that relay peripheral somatic and visceral nociceptive signals to the central nervous system (1–3). Genetic manipulation of DRGs through viral vectors has emerged as an essential core technique for decoding pain pathways, optogenetic/chemogenetic neuronal manipulation, and preclinical chronic pain gene therapy (4–8). Beyond pain research, DRG-targeted genetic manipulation also enables functional dissection of proprioceptive and exteroceptive afferent circuits for locomotion modulation and spinal-cord-injury-related motor recovery (9–11). The L3-L6 lumbar DRGs in laboratory mice have been employed in somatosensation of the hindlimb, neuropathic pain model construction, and study of abdominal pelvic visceral nociception (12–18).

Currently, three different strategies have been employed for gene transfection to DRGs, but each carries its own limitations. Intrathecal viral infusion leads to broad transduction of the spinal cord but suffer from poor selectivity for the DRG, with preferential labelling of larger diameter mechanoreceptors than the small nociceptive C-fibers (19, 20). Peripheral nerve injection relies on retrograde axonal transport but transduces different fibre populations inconsistently and preferentially targets motor over sensory neurons (21, 22). The need for very high titers of adeno-associated virus (AAV) following systemic administration stems from its DRG affinity influenced by type subtypes, thus producing a large interindividual variability (12, 23–25). Microinjection into the ganglion, in contrast, can yield cell-type-specific and segment-restricted transgenic expression without off-target neural labeling (20, 26–29). But even this intraganglionic approach has its own drawbacks. Traditional access to the DRG typically requires a sizable laminectomy or excision of the facet joints. Although this approach provides a wide exposure of the DRG, they come with several risks: including spinal instability, excessive postoperative tissue adhesions, and poor reproducibility across animals (21, 26, 30–34). To make the thing even worse, during microinjection, the spontaneous displacement of the DRG due to respiration and heartbeat induces severe damage of the neuronal cell body with the puncture needle (26, 28, 30). Moreover, while conventional spinal suspension holders can reduce respiratory-driven DRG displacement for single- or dual-segment injections in rodents, residual segment-level soft-tissue shift persists across extended lumbar operative fields. This local tissue movement can still cause neuronal injury during high-precision micro-injection targeting multiple contiguous lumbar ganglia. In addition, conventional methods of restricting injections to a single lumbar ganglion largely limit its application for studies of afferent signal convergence or segmental organization (26, 30). Published anatomical descriptions of mouse lumbar DRG innervation show inter-study discrepancies complicated by lumbosacral skeletal variants; furthermore, segmental innervation patterns differ between mice and rats, limiting direct cross-species extrapolation (35). Classic DRG microinjection protocols (26, 30)have not fully resolved these ambiguities, motivating our in-house anatomical validation to guide accurate multi-level targeting.

To address these challenges, we built our approach on several anatomical observations: the V-shaped white lines on the spinous processes as reliable vertebral landmarks, YFP-based mapping of segmental innervation patterns, and a detailed understanding of the DRG-facet joint spatial relationship. We therefore adopted a caudal-to-rostral counting strategy, engineered a custom spinal stabilizer (Supplementary file), and developed a protocol enabling accurate multi-segment (L3–L6) DRG viral injection with minimized bone resection and reduced motion-induced neuronal injury. To validate this workflow, we performed serial behavioral tests in mice receiving 1 to 8 DRG injections and and we confirmed functional viral transduction using air-puff-evoked *in vivo* GCaMP8s calcium imaging, with wild-type mice serving as negative controls.

## Materials and methods

### Animals and viral vectors

Male and female C57BL/6 J mice (C57 mice, *n* = 70) aged 8–10 weeks (body weight 20–26 g) were purchased from Charles River (China). Three transgenic mouse strains were exclusively used for anatomical mapping and surgical landmark verification: B6. Cg-Tg(Thy1-YFP)HJrs/J (Thy1-YFP-H, *n* = 5) mice (stock no. 003782, The Jackson Laboratory), which express yellow fluorescent protein (YFP) in primary DRG sensory neurons; B6.129P-Cx3cr1tm1Litt/J--1 (Cx3cr1-GFP, *n* = 5) mice (stock no. 005582, The Jackson Laboratory), which express green fluorescent protein in DRG-resident macrophages; C57BL/6 J-Tg(Thy1-GCaMP6s)GP4.3Dkim/J (Thy1-GCaMP6s, *n* = 5)mice (stock no. 024275, The Jackson Laboratory), which constitutively express GCaMP6s calcium indicator in a subset of DRG neurons for static anatomical fluorescence imaging. Animals were housed 4–5 per cage under standardized laboratory housing (22 ± 2 °C, 50 ± 10% humidity, 12 h light/dark cycle) with unlimited access to standard rodent chow and autoclaved drinking water. Animals were randomly assigned to experimental conditions, with age and sex balanced across groups. All animal-involved experiments were approved by the Animal Ethics Committee of China Pharmaceutical University and were conducted in accordance with the National Institutes of Health guidelines for the use of laboratory animals. The animal facility is accredited by the Association for Assessment and Accreditation of Laboratory Animal Care International (AAALAC).

Recombinant adeno-associated viral vector rAAV9-hSyn-jGCaMP8s-WPRE (catalog BC-0579, functional titer = 5.10 × 10^12^vg/mL, BrainCase, Shenzhen, China) was utilized for terminal *in vivo* functional calcium imaging transduction. The AAV9 serotype combined with neuron-specific hSyn promoter was selected for well-documented high expression efficiency in murine primary DRG neurons (23, 36).

### Vertebral and neural tissue preparation

Mice were euthanized via an overdose of isoflurane (5% for induction, 3% maintenance until respiratory arrest; death verified by loss of the toe-pinch reflex). Animals were positioned prone, and a midline skin incision was created extending from the T12 vertebral level (localized based on the lowest rib, T13) to the base of the tail, with bilateral extensions toward the hindlimbs. The skin was retracted by blunt and sharp dissection to expose the underlying fascia. The vertebral column was transected at T11-T12 and caudal vertebra base using bone rongeurs. Hindlimbs were amputated below the knee; pubic symphysis was sharply cut to open the abdominal cavity for visceral organ removal. Surrounding muscle tissue was roughly trimmed, while tissue surrounding intervertebral foramina and nerve tracts was fully preserved intact. Dissected vertebral blocks were fixed in 4% paraformaldehyde (PFA) at 4 °C for 4–6 h. Transcardial perfusion was omitted to retain native perivascular connective tissue architecture surrounding DRGs. Specimens were transferred to PBS and stored overnight at 4 °C before downstream anatomical processing.

Sequential dorsal laminectomy from T13 to L6 was performed on fixed specimens to expose lumbar spinal cord and bilateral L1-L6 DRGs housed within intervertebral foramina, each wrapped by thin translucent connective capsule. L1 level was confirmed via T13 rib counting. Bilateral pedicles were removed; a micro-neural dissector separated spinal cord and DRGs along the plane between posterior longitudinal ligament and spinal dura mater. Spinal nerves were traced peripherally until all rostral intersegmental fiber connections terminated, then sharply cut. The sciatic nerve was dissected distally along the femur posterior surface toward the popliteal fossa, where it trifurcates into tibial, common peroneal and sural branches. The nerve was transected 1–2 mm distal to the trifurcation, and the full trunk harvested. Isolated nerve tissue was flattened on glass slides for brightfield or fluorescence imaging, with continuous PBS irrigation to prevent tissue drying.

### Experimental group design

To evaluate the potential damage of the injection procedure, sixty C57BL/6 J mice were randomly divided into ten experimental groups (*n* = 6 per group). Groups 1–8 received microinjection of viral vectors into different combinations of lumbar DRGs, including left L4 (L4), left L4-L5 (L4-5), left L3-L5 (L3-5), left L3-L6 (L3-6), bilateral L4 (Bi-4), bilateral L4-L5 (Bi-L4-5), bilateral L3-L5 (Bi-L3-5), and bilateral L3-L6 (Bi-L3-6) DRGs, respectively. Group 9 served as the sham group, in which mice underwent identical surgical exposure and spinal stabilization without facet joint-guided drilling or viral injection. Group 10 was designated as the naïve group with no surgical intervention or treatment. Behavioral assessments were conducted at the baseline preoperative time point and on postoperative days 3, 5, 7, 14, and 21.

Sample size was estimated *a priori* based on pilot data (effect size 0.8, α = 0.05, power = 0.80), yielding 5 mice per group; to account for potential attrition, 6 mice per group were used (total *n* = 60). Mice were randomly assigned using a computer-generated sequence, with allocation concealed in sealed opaque envelopes. The surgeon was not blinded, but behavioral testing and offline analysis were performed by two independent investigators blinded to group allocation. Exclusion criteria included persistent bleeding >2 min, wound infection, weight loss >20% within 3 days, or hindlimb paralysis; none were met. Both male and female mice were used in balanced numbers (3 per sex per group), and female tests were scheduled at the same estrous phase to control for hormonal effects.

### Quantitative behavioral assessments

All behavioral tests were performed between 9:00 a.m. and 4:00 p.m. Mice were acclimated to the testing room for a minimum of 30 min before each testing session. All equipment was wiped with 75% ethanol and fully air-dried between animals to reduce olfactory confounding cues.

#### Body weight measurement

Whole-body mass was measured at all time points using a 0.01 g precision digital balance to track general postoperative health status.

#### Von Frey test

Mechanical nociceptive sensitivity of both hind paws was evaluated with an Aesthesio® Precision Tactile Sensory Evaluator (DanMic Global, LLC, San Jose, CA, USA) equipped with calibrated monofilaments spanning 0.008–2.0 g (37). Mice were habituated for 60 min inside transparent Plexiglas chambers positioned on an elevated wire mesh grid. The up-down method was adopted to calculate the 50% paw withdrawal threshold (PWT) for each hind paw (38). Briefly, a monofilament was applied perpendicularly to the plantar surface of the hind paw for 1–2 s with enough force to induce mild bending. An abrupt paw withdrawal or flinch was regarded as a positive response. Testing started using the 0.16 g filament, and filament selection followed the up-down algorithm. Stimuli were separated by an interval of no less than 10 s to prevent sensory sensitization. The left and right hind paws were tested alternately, and withdrawal thresholds were documented independently.

#### Rotarod test

Motor coordination balance was assessed on a rotarod apparatus (Model KW-6C; KEW, Nanjing Kew Biotechnology Co., Ltd., Nanjing, China). Mice were given adaptive training for three days before baseline acquisition. Animals conducted three 60 s trials at a fixed speed (4 rpm) every training day. They also made three trials (4–40 rpm within 5 min) under acceleration mode (31). During formal testing, the rod linearly accelerated from 4 rpm to 40 rpm for 5 min with the latency to fall recorded with a cutoff value of 300 s. The average duration of time taken to fall down by mouse in all the three trials was used for statistical analysis. Each mouse completed 3 trials per test session separated by ≥15 min.

#### Open field test

Locomotor activity observed spontaneously in square open field arenas (40 × 40 × 40 cm) in low-level ambient light in environment. Each mouse was placed at the center of the arena and allowed to explore freely for 10 min. The software MouseScope Desktop (v3.4.0) tracks and quantifies locomotion. Investigated variables encompassed path of travel, ranging of travelling speed, average speed and the top speed. Top speed for each species was defined as the average of all velocity data points greater than the 75th percentile of its speed distribution. The arena was cleaned between trials to remove scent cues.

### Statistical analysis

Sample size was determined by *a priori* power analysis (effect size = 0.8, α = 0.05, power = 0.80; *n* = 6/group allowing for attrition). Normality was assessed via Shapiro–Wilk test. Outliers were defined as data points exceeding ±3 standard deviations from group mean; no animals met outlier exclusion criteria in the present dataset per pre-specified exclusion thresholds. Mixed-design repeated-measures ANOVA (between-factor: Group; within-factors: Time, and Side where applicable) was used for behavioral data, with Greenhouse–Geisser correction when sphericity was violated. For post-hoc pairwise comparisons, we focused on pre-planned contrasts (within-group comparisons against baseline across time points, and group comparisons at selected key time points). To control for multiple testing while avoiding excessive conservatism due to the large number of potential contrasts, the Benjamini–Hochberg False Discovery Rate (FDR) procedure was applied at a q-value of 0.05. Paired t-tests were used for within-group comparisons against baseline, followed by FDR correction. Cross-sectional group comparisons at single time points used one-way ANOVA with FDR-corrected post-hoc tests. Terminal calcium imaging data were evaluated qualitatively without quantitative statistics. All data are mean ± SEM; significance threshold *p < 0.05, **p < 0.01, ***p < 0.001 (FDR-adjusted). Analyses used Python 3.11 and GraphPad Prism 10.1.2.

## Results

### Vertebral landmark identification

Anatomical landmark validation was performed using wild-type C57BL/6 J mice (*n* = 6) and three C57BL/6 J-background transgenic lines: Thy1-YFP-H (*n* = 3), Cx3cr1-GFP (*n* = 3), and Thy1-GCaMP6s (*n* = 3). After midline dorsal skin cut and lateral skin flap retraction, spinous process white lines(thickened superficial thoracolumbar fascia adjacent to spinous processes overlying the longissimus muscle) became macroscopically visible. In rare instances where lumbosacral adipose tissue obscured the landmark, superficial fat was gently blunt-dissected away; tissue desiccation blurring the white line was reversed with saline-soaked cotton swabs for rehydration. At each vertebral level, the white line forms a bilaterally symmetric V-shape extending cephalolaterally from the spinous process on both sides (Figure 1A). The most caudal V-shaped line corresponds to the L6 spinous process, where a 5–0 non-absorbable suture was placed as a landmark at the inferior margin (Figure 1B). Vertebrae were counted sequentially rostrally starting at L6: L6 → L5 → L4 → L3 → L2 → L1.

**Figure 1:**
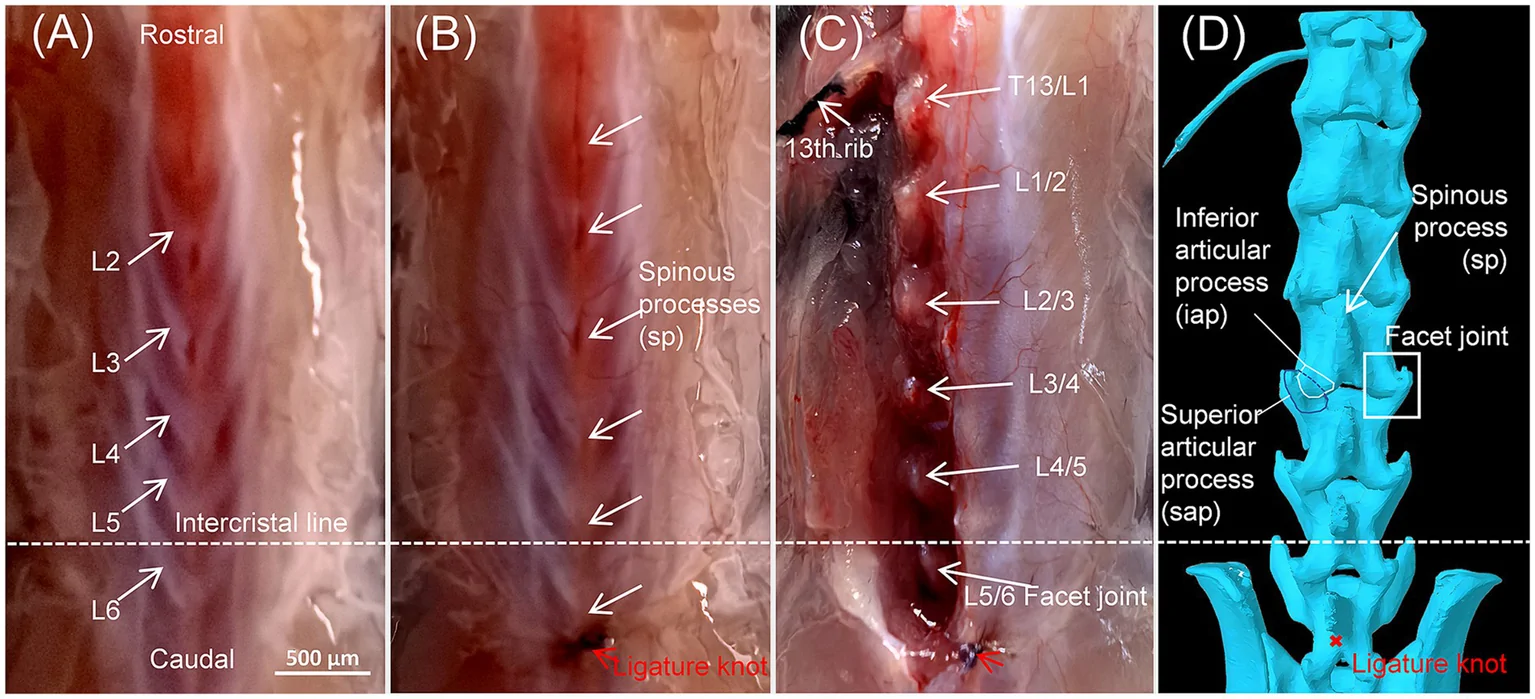
Anatomical validation of spinous V-shaped white line landmarks for reproducible lumbar vertebral localization (*n* = 15). **(A)** Microscopic examination of white dorsal macropsopic structures in L2-L6 spines. **(B)** A permanent, non-absorbable, ligature knot is firmly attached to the inferior margin of the spinous process of L6 to allow for a stable identification of the corresponding vertebrae in the first surgery and for the re-examination on postoperative day 21. **(C)** After a partial resection of the paraspinal muscles, the 13th rib is revealed and the facet joints are labelled sequentially from T13/L1 to L5/L6. **(D)** Micro-CT 3D reconstruction of lumbar bony landmarks, spinous processes, superior articular processes, inferior articular processes, and facet joints, with fixed ligature marker on L6. The abbreviations of sp., sap, and iap refer to spinous, superior, and inferior articular processes, respectively, and micro-CT micro-computed tomography. White line: thickened superficial thoracolumbar fascia adjacent to spinous processes overlying the longissimus muscle.

To validate the accuracy of this white-line-based counting, the 13th ribs of all mice were identified after removal of the left paraspinal muscle tissue, employing the standard rib-counting technique (35) (Figure 1C). In addition, three-dimensional models were utilized to visually illustrate the correspondence between the white line landmarks and the underlying bony structures (Figure 1D).

### Mapping of lumbar DRG innervation

To trace axonal trajectories and intersegmental nerve connectivity, brightfield and epifluorescence imaging were performed using an EMS3/SyCoP3 microscope on intact lumbar spinal cord and ipsilateral peripheral nerve tracts from Thy1-YFP-H mice (*n* = 5, including 3 from the cohort described above and 2 additional mice) to trace axonal trajectories and intersegmental nerve connectivity (Figure 2A). YFP axonal tracing showed that L1-L2 segments did not contribute to the sciatic nerve, but together with some nerve fiber branches from L3, formed the obturator and femoral nerves (Figure 2A1); the nerve fiber bundles from L3-L5 segments formed the sciatic nerve (Figure 2A2); and L6 segments, instead of participating in the sciatic nerve, together with small branches from L5, formed the pudendal nerve (Figure 2A3).

**Figure 2:**
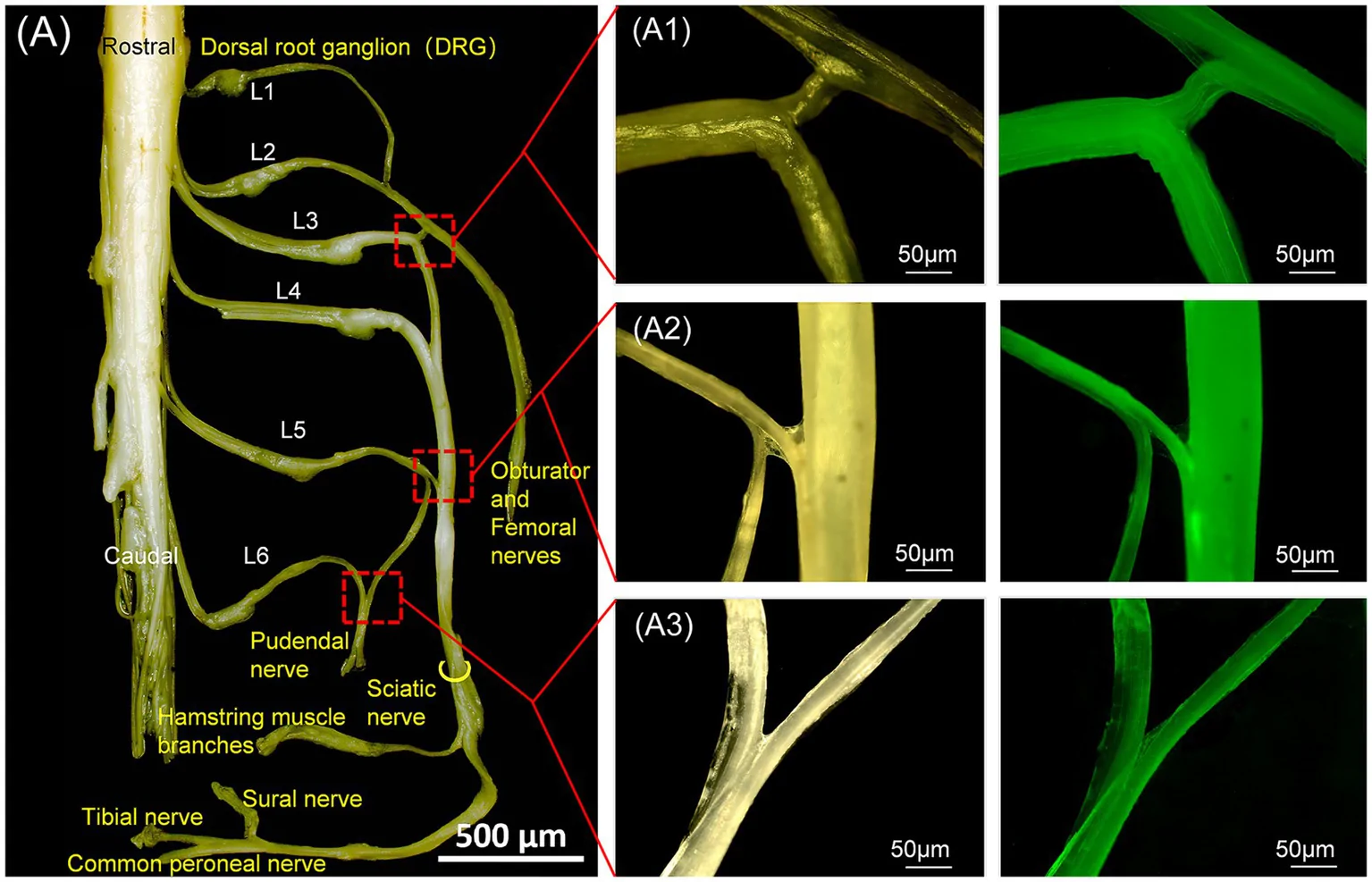
The segmental topography of the neural fibers of the lumbar dorsal root ganglia traced by yellow fluorescent protein YFP in Thy1-YFP-H mice (*n* = 5). **(A)** General view of lumbar spinal cord and lumbosacral nerve plexus (rostral = top, caudal = bottom). Three key nerve convergence regions were marked through red dashed boxes (Scale bar = 500 μm). Paired magnified panels (**A1–A3**) show brightfield (left) and YFP fluorescence (right) micrographs, scale bar = 50 μm. **(A1)** Partial L3 axons connect with L1 and L2 nerve trunks to create obturator and femoral nerves, while L1 and L2 fibers do not participate in the sciatic nerve. **(A2)** The L5 spinal nerve splits into two branches: one branch joins the nerve trunk formed by L3 and L4 fibers to constitute the sciatic nerve. **(A3)** The L6 spinal nerve, together with a small branch originating from L5, forms the pudendal nerve and does not participate in the composition of the sciatic nerve.

To further validate these segmental innervation patterns, we performed anatomical dissection and brightfield examination of the lumbosacral plexus in all mice from the vertebral landmark identification and facet osseous landmark-DRG spatial correspondence experiments (total *n* = 20), which included wild-type C57BL/6 J (*n* = 10), Cx3cr1-GFP (*n* = 5), and Thy1-GCaMP6s (*n* = 5) mice. The nerve trunk compositions and segmental contributions observed in these additional mice were entirely consistent with the YFP-based tracing results, confirming that the described innervation topography is not restricted to the Thy1-YFP-H strain.

### Facet joint landmark-DRG spatial correspondence

Based on anatomical descriptions in previous literature (26, 31), we generated a schematic illustration of the peripheral anatomy of the DRG, showing its spatial relationship with the facet joint. Of note, the DRG is not positioned immediately beneath the facet joint; rather, it resides at a more rostral position relative to the joint (Figure 3A). This arrangement underlies our ability to achieve DRG viral injection without damaging the facet joint. On lumbar vertebral specimens from Cx3cr1-GFP mice, using the facet joints as anatomical landmarks, the drilling coordinates were pre-marked at the base of the inferior articular process (Figure 3B, left); holes were prepared using a micro-bone drill (Figure 3B, middle). Markers were added to the facet joints and drilling sites in the 3D CT reconstruction model to visually illustrate the spatial relationship between the drilling sites and the facet joints (Figure 3B, right).

**Figure 3:**
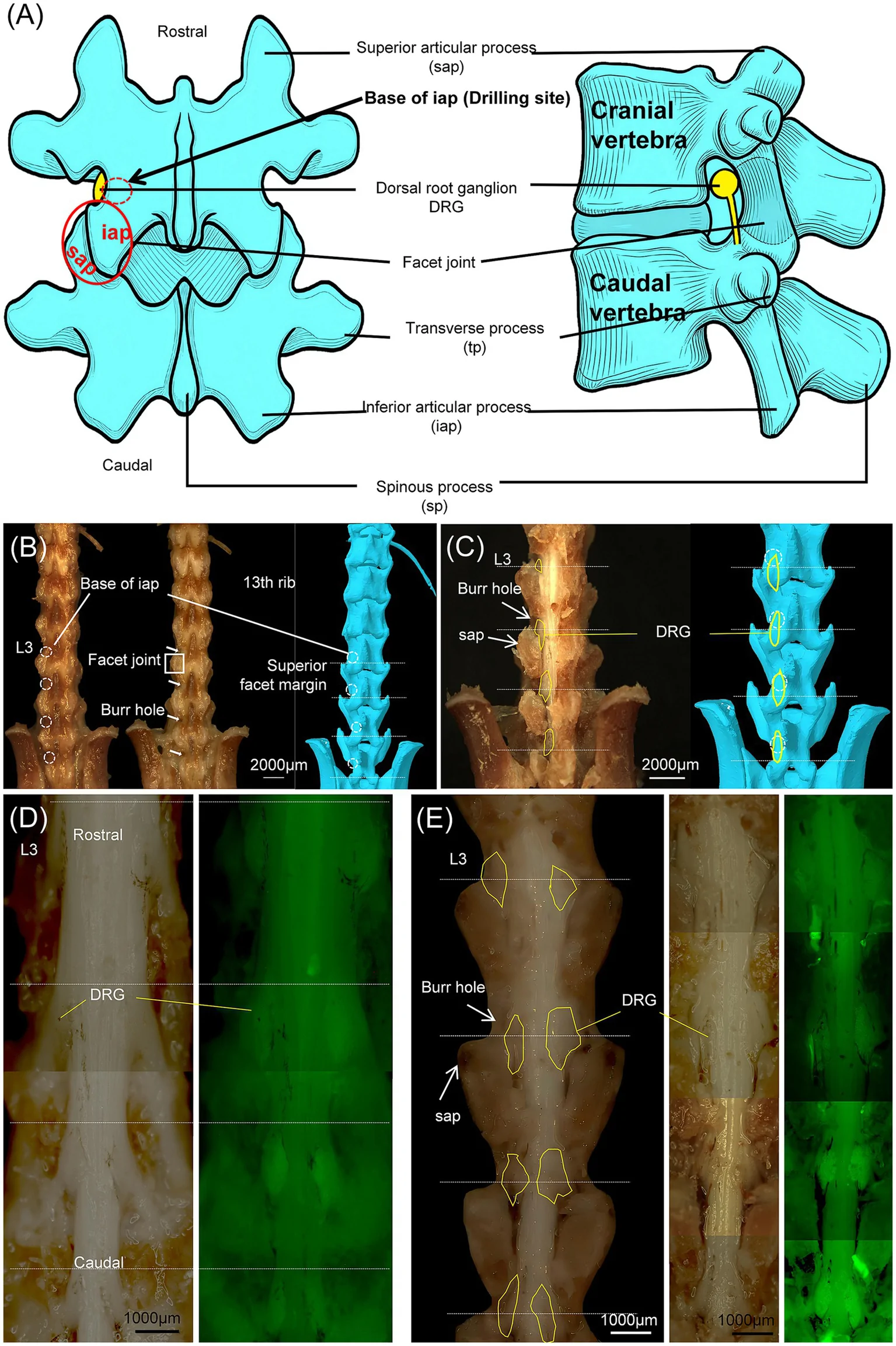
Spatial anatomical matching between L3–L6 DRGs and lumbar facet joints for standardized drilling coordinate validation. This figure illustrates the spatial anatomical correspondence among lumbar vertebrae, facet joints, and target L3-L6 DRGs. Drilling sites are positioned at the base of the inferior articular process of the cranial vertebra within each facet complex, with a drilling trajectory oriented toward the underlying DRG. **(A)** Schematic illustration of the peripheral anatomy of the dorsal root ganglion (DRG). This posterior-left view of the spine shows the spatial relationship of the DRG with adjacent structures. **(B)** Note the dashed circled areas that represent sites of burr holes at the bottom of each iap (inferior articular process) in this lumbar bone specimen (left). Post microdrilling gross anatomy (middle) Micro-CT 3D reconstruction (right) visualizing the vertical alignment of burr holes ((0.5 mm in diameter, drilled to the depth of the bone thickness)), facet joints, sap and underlying DRGs. Scale bar = 2000 μm. **(C)** The image (left) shows a unilateral partial laminectomy with yellow outlines demarcating DRG projections underneath the drilling sites. Corresponding micro-CT reconstruction (right); scale bar = 2000 μm. **(D)** A thorough bilateral laminectomy that exposes the spinal cord and preserves the DRGs; a composite brightfield/fluorescence image of a Cx3cr1-GFP mouse; scale bar = 1,000 μm. **(E)** The smooth and intact facet samples obtained from Thy1-GCaMP6s mice are given. Yellow lines demarcate the boundaries of the DRG, demonstrating the anatomical correspondence between the DRG and the facet joint in this specimen. Scale bar = 1,000 μm.

Part of the drill hole marks was retained, and the left lamina was removed to expose the deep DRG (Figure 3C, left). The contour line connecting the facet joint to the DRG was traced (Figure 3C), confirming that each DRG was located directly below its corresponding drill hole site. After completely resecting all laminae while preserving intact superior articular processes, the bilateral dorsal root ganglia and the spinal cord can be observed under brightfield and fluorescence illumination (Figure 3D).

For Thy1-GCaMP6s mouse specimens, the facet joints are kept intact throughout the procedure, and the exposed areas of the dorsal root ganglia are circled, clearly demonstrating the stable anatomical correspondence between the two (Figure 3E).

To validate this spatial relationship across genotypes, we examined wild-type C57BL/6 J (*n* = 10, including 6 from the landmark identification cohort and 4 newly added), Cx3cr1-GFP (*n* = 5, including 3 from the cohort and 2 new), and Thy1-GCaMP6s (*n* = 5, including 3 from the cohort and 2 new) mice (total *n* = 20). Landmark-based drilling reliably exposed target DRGs, identifiable via grayish-white gross appearance and surrounding vasculature.

### Surgical procedure

#### Anesthesia and preoperative preparation

Mice received intraperitoneal tribromoethanol (250 mg/kg) for initial surgical dissection. Continuous isoflurane inhalation (1–1.5% in oxygen, flow rate 1 L/min) via nose cone maintained anesthesia during viral injection. Adequate anesthetic depth was verified via complete loss of toe-pinch reflex. Ophthalmic gel was applied bilaterally to avoid corneal desiccation under prolonged anesthesia.

#### Step 1–2: depilation and midline skin incision

Thoracolumbar dorsal skin was fully depilated with rodent hair removal cream (Figure 4A). Iliac crests and spinous processes were palpated to mark superficial landmarks, a cross-shaped reference drawn on skin (Figure 4B). A 2.5-cm longitudinal midline cut was made along the reference line (Figure 4C). V-shaped spinous white lines were identified; 5–0 non-absorbable polypropylene suture tagged the inferior L6 spinous margin as a permanent vertebral counting marker.

**Figure 4:**
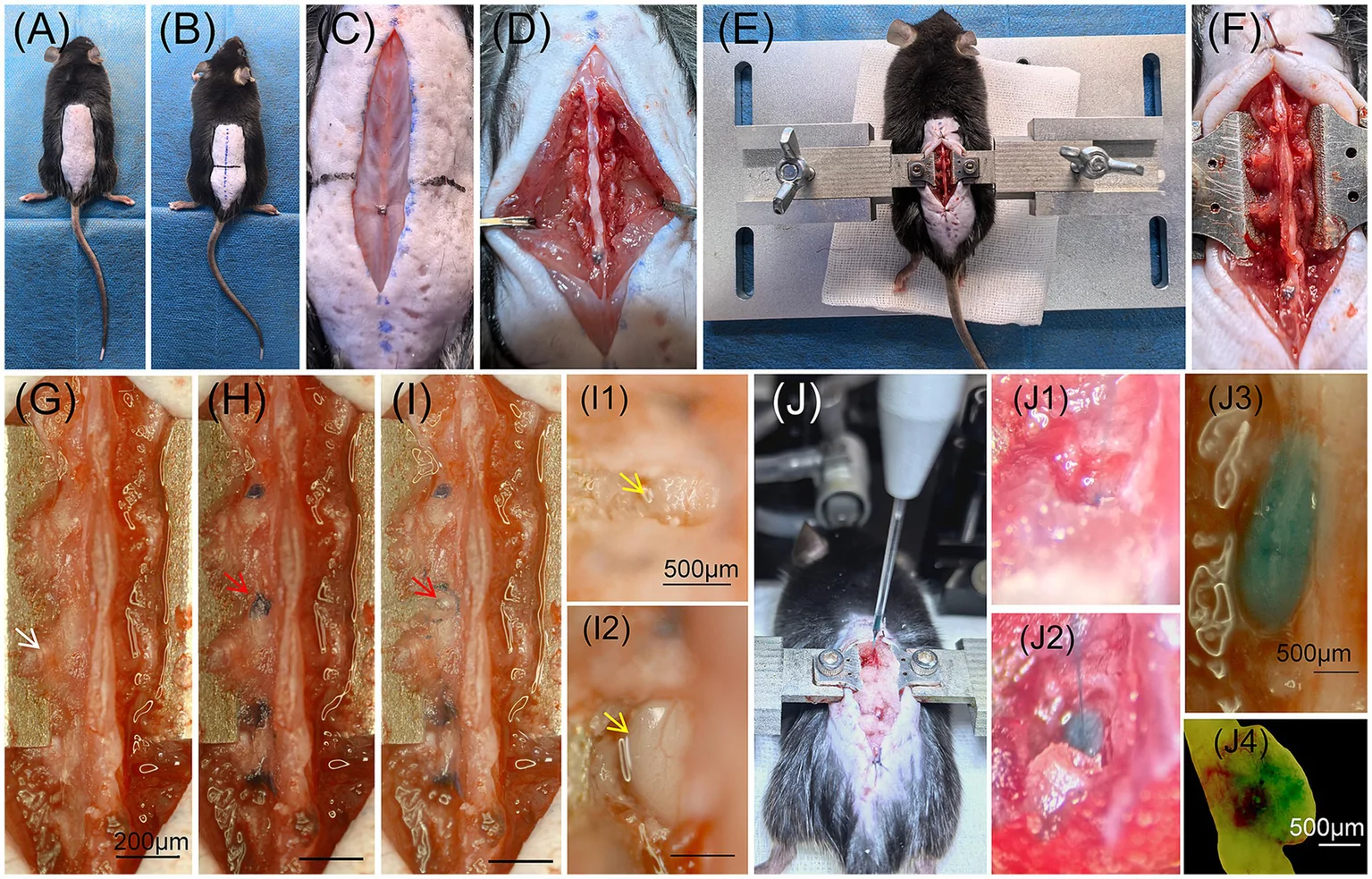
Surgical pipeline for multi-segment lumbar DRG recombinant adeno-associated virus (rAAV) microinjection. **(A–C)** Preoperative preparation: **(A)** Skin depilation of Thoracolumbar dorsal skin; **(B)** Cross-reference skin marks were tattooed at the iliac crest and the spinous process of the palpated vertebra; **(C)** A 2.5 cm midline incision was performed with the permanent 5–0, non-absorbable suture anchored at the inferior margin of the L6 spinous process. **(D)** Bilateral dissection of the paraspinal muscles for full facet joint exposure intraoperatively bony references. **(E,F)** The custom-made spinal stabilizer prevents displacement of the DRG due to respiratory/cardiac pulsations and redundant skin is partially sutured to create a stable surgical window. **(G–I)** Targeted microdrilling performed at the base of the inferior articular process of the cranial vertebra of each facet pair, which serves as the consistent drilling coordinate for L3–L6 DRG access. Burr holes were made with a 0.5-mm drill bit, advanced through the lamina (0.5–0.8 mm per level) until a sudden loss of resistance indicated the endpoint. **(I1,I2)** Magnified micrographs of specimens with intact periosteum and resected periosteum, scale bar = 500 μm. **(J)** Co-injection of rAAV9-hSyn-jGCaMP8s with Fast Green tracer (0.5% w/v in sterile saline) into the DRG. A glass micropipette (tip outer diameter ~30 μm) was advanced to a depth of approximately 0.3 mm below the DRG capsule surface for each injection; **(J1,J2)** Sequential images illustrating tracer diffusion within the DRG capsule during the injection; **(J3)** *In situ* verification post-laminectomy. **(J4)** Ex vivo macroscopic view of the injected DRG. For every DRG, a volume of 200 nL of viral vector was injected at a rate of 40 nL/min. Further, each injection was followed by a 5-min dwell time before the retraction of the pipette to prevent the backflow of the viral vector.

#### Step 3–4: muscle dissection and spinal stabilization

Bilateral paraspinal muscles were sharply cut and retracted laterally to fully expose facet complexes as osseous landmarks (Figure 4D). Custom spinal stabilizer was mounted on stereotaxic frame to rigidly immobilize the vertebral column and eliminate respiratory/cardiac DRG shift (Figures 4E,F). Redundant incision skin was partially sutured with 5–0 absorbable polyglactin to construct a stable central surgical window.

#### Step 5: facet joint landmark-based targeted microdrilling

The position for drilling at levels L3-L6 was defined using the facet joints, which were found to be consistent bony references. The position was fixed at the base of the inferior articular process of the upper vertebra of the facet pair. Small bony windows were created at all pre-marked landmarks using a 0.5-mm high-speed micro-drill, with the endpoint determined by a sudden loss of resistance upon penetrating the inner cortical bone. (Figures 4G–I). After drilling, the cover was a gelatine sponge wet with saline. At every drill site, two periosteum states were recorded: intact periosteum, which completely covered the DRG capsule, or surgically resected periosteum (Figures 4I1,I2). If it is unclear whether the DRG lies beneath the periosteum, the periosteum can be removed to confirm exposure of the DRG; for experienced operators, the periosteum may be preserved to avoid the risk of damaging the DRG during removal.

Anatomically, each L3-L6 target DRG is positioned rostral relative to its paired facet-joint complex. The drilling burr site is placed at the base of the inferior articular process belonging to the cranial vertebra of each facet pair, generating a near-vertical bony access trajectory toward the underlying DRG. Owing to the small lumbar skeletal dimensions in mice, absolute millimetric distances from the bony drilling surface to the DRG exhibit inter-specimen variability; accordingly, landmark-based positioning (26, 30, 31) rather than rigid fixed distance values serves as the primary practical reference for operators.

#### Step 6: viral/tracer microinjection

Platform of anesthetized mice was secured to stereotaxic micromanipulator with a constant isoflurane supply. Before the injection, the drill holes were covered with gelatin sponge, which was removed. To verify the accuracy of landmark-guided injections, Fast Green dye was co-injected. Upon initiation of the injection, the dye swiftly diffused throughout the DRG capsule (Figures 4J–J2). After the injection and optional resecting periosteum, *in situ* lateral imaging and ex vivo imaging confirmed complete filling of the capsule by tracer (Figures 4J3,J4).

The needle was advanced vertically until its tip contacted the DRG surface, at which point the Y-axis coordinate was set to zero. Continued advancement produced a visible surface depression; a sudden restoration of this depression indicated that the needle had pierced the DRG capsule. The needle was then advanced an additional 0.2 mm. At this stage, either of the following criteria was met before initiating virus injection: (i) the needle was retracted until the surface depression was completely restored, or (ii) if the total depth from the original surface contact (Y = 0) had reached 0.45–0.5 mm, no retraction was performed. Inject 200 nLs (26) into each DRG at a delivery speed of 40 nL/min using a pulled glass micropipette (~30 μm tip outer diameter). The pipette was held in position for 5 min after the injection before slowly withdrawing it to avoid leakage due to viral backflow.

#### Step 7: wound closure and postoperative care

Before suturing, the site should be thoroughly irrigated with sterile saline and gentle pressure used to control residual bleeding. Displace the bilateral paraspinal muscle and then carry out interrupted midline suturing on the spinous process with 5–0 absorbable suture. (Figure 5A). After the operation, ampicillin was given to drinking water for 3 successive days for infection control and ibuprofen was given for pain relief. The surgical incision was monitored for its healing. Skin pigmentation and hair growth appeared between 5 and 7 days post-operation. After 21 days of the operation, the hair in the operating wound was almost at its normal appearance (Figure 5B). Macroscopic assessment was performed at 21 days post-surgery. Evaluation at this time showed the spinal cord and dorsal root ganglia to be smooth and free from tissue adhesion. The vertebral laminae were intact without compressive damage. Slight drilling effect was seen at only a few of the lumbar segments (Figure 5C).

**Figure 5:**
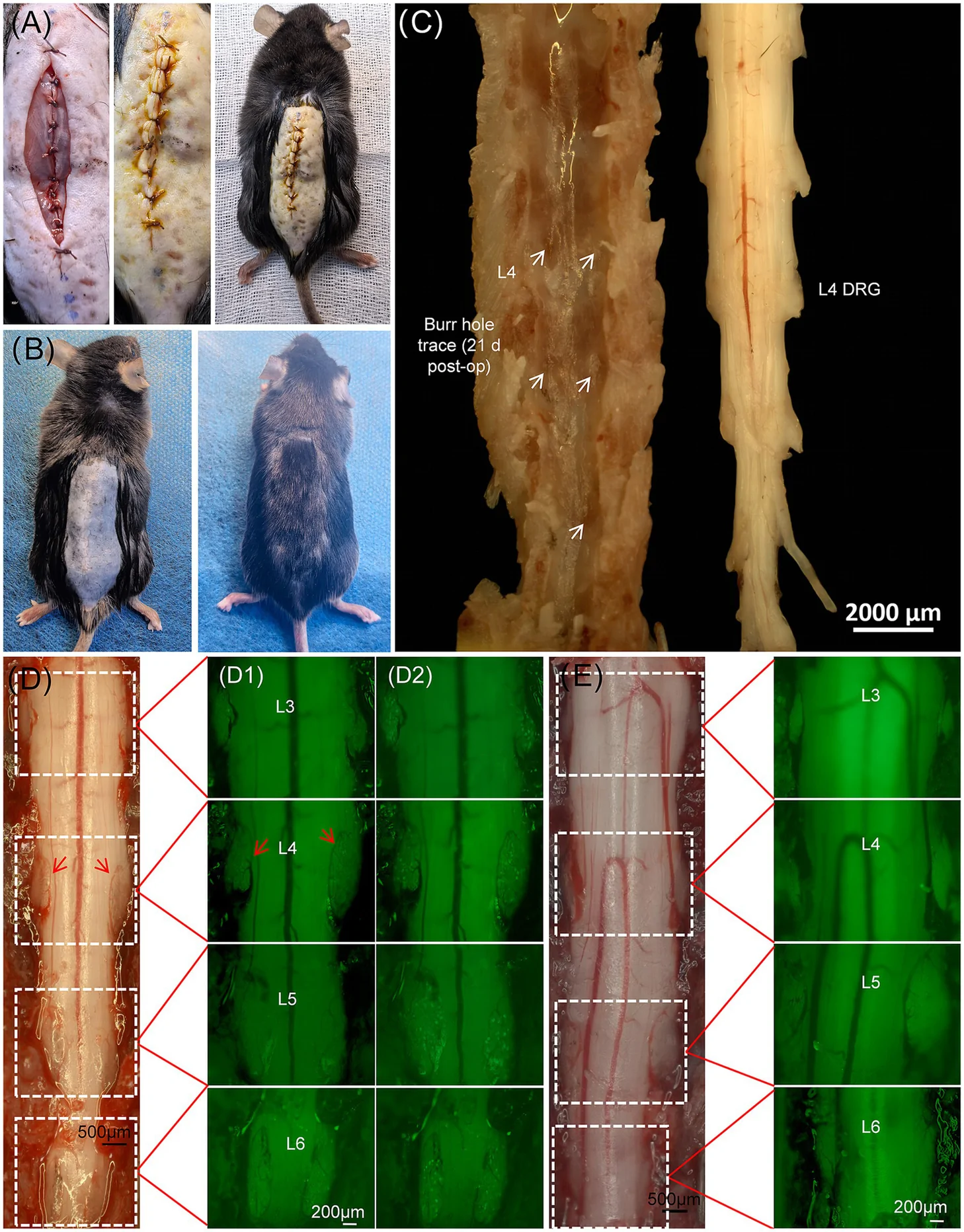
Postoperative wound healing and viral transduction verification (POD 21 *in vivo* calcium imaging). Calcium imaging was performed in 48 virus-injected, 3 sham-operated, and 3 naïve mice. In total, 432 DRGs were imaged; stimulus-evoked calcium transients were detected in all 180 virus-injected DRGs. **(A)** A layered closure is used intraoperatively in repositioning the paraspinal muscles followed by interrupted sutures to the midline and skin. **(B)** POD 5–7 (left) shows cutaneous pigmentation; POD 21 (right) exhibits nearly full hair regrowth. **(C)** POD 21 has faint residual drill traces indicated by white arrows. The spinal cord and DRG surfaces were smooth and did not have any adhesion. The vertebral laminae were intact. Scale bar = 2000 μm. **(D)** *In vivo* calcium recordings from the bilateral L3-L6 DRGs of virally-injected mice. **(D1)** Representative static snapshot of DRG fluorescence under anaesthesia without peripheral stimulation; no detectable stimulus-associated calcium signal was observed prior to air-puff stimulation. **(D2)** Representative static snapshot captured during air-puff stimulation of the hind limb and perineum, showing robust local fluorescence elevation within the DRG. **(E)** Representative static snapshot from age-matched wild-type C57BL/6 J negative control mice acquired under identical imaging and stimulation conditions. No stimulus-triggered fluorescence elevation was observed in the DRG.

#### Terminal *in vivo* calcium imaging (POD 21)

On postoperative day (POD) 21, *in vivo* calcium imaging experiments were conducted under anesthesia. Calcium imaging was performed in all virus-injected mice (*n* = 48), as well as 3 sham-operated and 3 naïve control mice. For each animal, bilateral L3-L6 DRGs (8 DRGs per animal, 432 imaged DRGs in total across all imaged animals) were imaged. Stimulus-evoked calcium transients were observed in all 180 virus-injected DRGs within this imaged dataset. Bilateral L3-L6 dorsal root ganglia were exposed using the caudal-to-cranial vertebral counting method; non-absorbable sutures fixed to the lower margin of the L6 spinous process prior to surgery served as a stable anatomical reference, and the procedure was consistent with the previously described surgical protocol. Time-series images were acquired continuously to monitor stimulus-evoked calcium transients. All imaging recordings were acquired using a Zeiss microscopy system (EMS3/SyCoP3) equipped with a PlanApo Z 0.5 × objective (1.8 × zoom), an HXP-120 light source, and a Zeiss Axiocam 503 camera. For representative still images presented in the figures (Figures 5D–D2), a longer exposure time of 4.4 s was used to obtain high signal-to-noise ratio snapshots. Imaging parameters were set as an excitation wavelength of 493 nm, an emission wavelength of 517 nm, and an exposure time of 4.4 s. Gentle air-puff stimulation was delivered to the hindlimbs and perineal region. Age-matched C57BL/6 J wild-type mice were imaged under identical conditions as blank controls. Brightfield overview images of exposed bilateral L3-L6 DRGs were captured (Figure 5D). Under anesthesia and in the absence of peripheral stimulation, DRG neurons exhibited minimal spontaneous calcium activity (Figure 5D1). In contrast, air-puff stimulation targeting the hindlimbs and perineum reliably evoked robust calcium transients in DRG neurons (Figure 5D2). Under identical imaging and stimulation paradigms, no obvious calcium responses were detected in C57BL/6 J wild-type mice (Figure 5E).

Of note, the macroscopic wide-field imaging system used in this work enables qualitative assessment of stimulus-evoked calcium transients across whole DRG regions, but it does not achieve single-neuron resolution. Rigorous quantitative calcium metrics including ΔF/F amplitude, fraction of responsive neurons, and response kinetics require two-photon microscopy setups to resolve individual neuronal somata, which cannot be derived by post-hoc processing of our current wide-field imaging dataset.

### Body weight displays group- and time-dependent recovery trajectories

Baseline body weight did not differ significantly among the ten groups [one-way ANOVA, F (9,50) = 1.24, *p* = 0.291], indicating successful randomization. However, a slight numerical difference was observed, which we attribute to the unavoidable minor variation in sex distribution. To account for this, subsequent analyses of body weight were performed using percentage change from individual baseline values.

A two-way mixed ANOVA on the percentage change data revealed significant main effects of time [Greenhouse–Geisser corrected *F*(3.15, 157.72) = 152.41, *p* < 0.001] and group × time interaction [*F*(28.39, 157.72) = 5.70, *p* < 0.001], while the main effect of group was not significant [*F*(9,50) = 1.06, *p* = 0.406], indicating that the overall recovery trajectories were similar across groups when baseline differences were controlled. Naïve mice showed a steady weight gain throughout the study. Sham animals exhibited a slight transient fluctuation but recovered rapidly. Within the first week after surgery, all surgical groups showed an initial decrease in body weight. From POD 7 onward, bilateral multi-segment groups displayed a strong rebound, and by POD 21 most groups had returned to baseline or above (Figure 6). Paired t-tests against baseline (FDR-corrected) confirmed a significant weight loss on POD 3–7 in all surgical groups.

**Figure 6:**
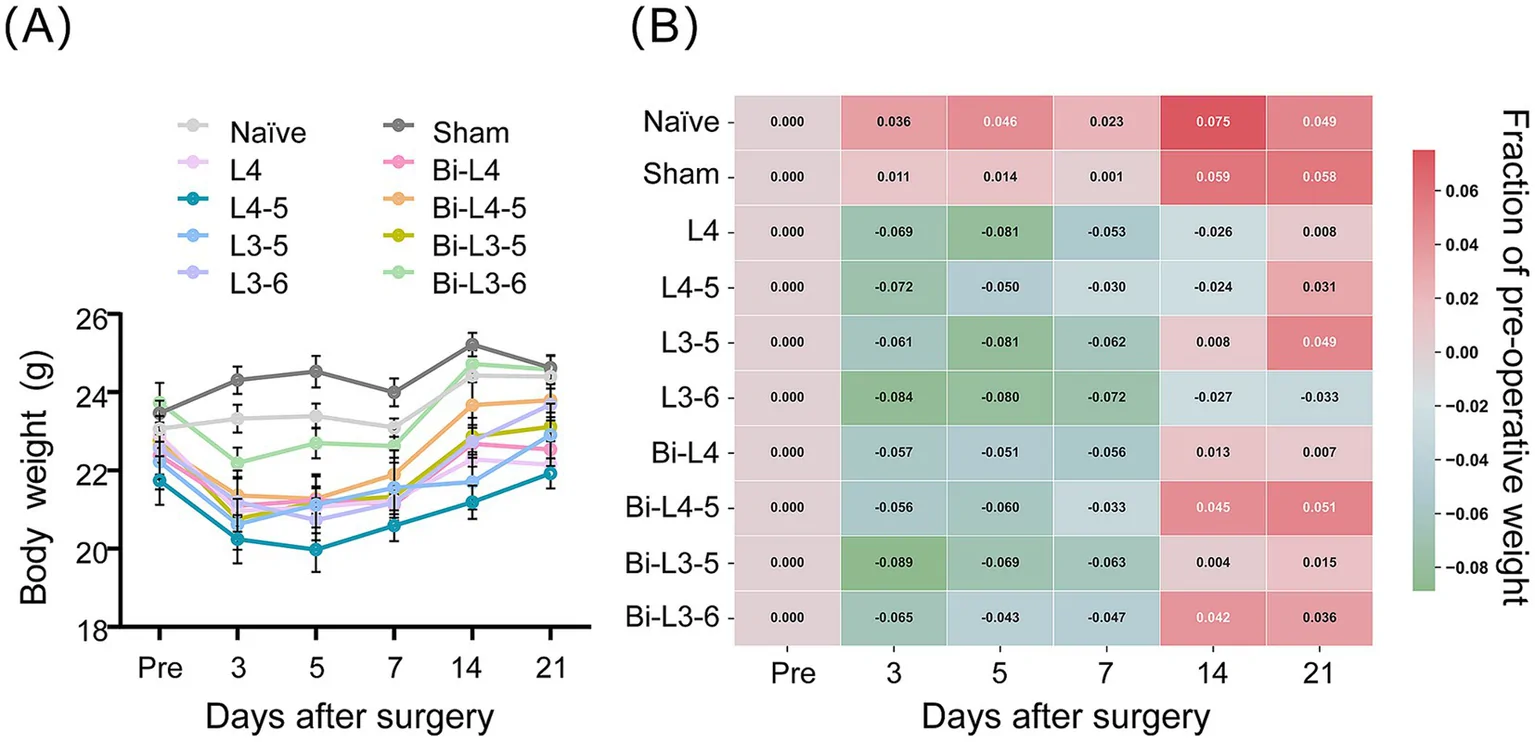
Mouse longitudinal body weight changes after DRG viral injection. Ten groups of animals were created (*n* = 6/group) comprising eight unilateral/bilateral DRG injection groups at different lumbar segments, sham group and naïve control. **(A)** Serial body weight expressed as mean ± SEM. All surgical groups exhibited a transient weight loss by POD 7. The bilateral multi-segment groups demonstrated a delay in weight recovery. By POD 21, the overall recovery trend was favorable, with most groups approaching baseline levels. **(B)** Heatmap showing relative body weight change expressed as a fraction of preoperative weight. Negative values denote weight loss relative to baseline, while positive values indicate weight gain.

### Bilateral injection induces reversible segment-dose mechanical hypersensitivity

To rule out mirror-image contralateral allodynia, we first compared ipsilateral versus contralateral PWT values across all surgical groups. A three-way repeated-measures ANOVA (side x group x time) revealed no significant main effect of side [*F*(1,50) = 0.52, *p* = 0.475] and no significant side x group [*F*(9,50) = 0.89, *p* = 0.541] or side x time [*F*(5,250) = 1.13, *p* = 0.344] interactions, confirming that unilateral injections did not produce detectable contralateral mechanical sensitization. Subgroup stratified three-way ANOVA was additionally performed within unilateral injection cohorts, confirming no subtle ipsilateral-to-contralateral mirror allodynia even in single-side DRG injection groups, supporting averaging of bilateral paw withdrawal thresholds. Consequently, left and right paw thresholds were averaged per mouse for all subsequent analyses.

Baseline PWT was uniform across all ten groups [one-way ANOVA F (9,50)=1.480, *p* = 0.181]. Two-way repeated-measures ANOVA identified significant main effects of group [*F*(9,50)=7.260, *p* < 0.001], time [*F*(5,250) = 12.830, *p* < 0.001], and interaction [*F*(45,250) = 4.910, *p* < 0.001] (Figure 7). Sham mice only displayed mild, transient threshold reduction POD5 [28% drop vs. baseline; POD5 t (5)=3.124, *p* = 0.026], fully normalized POD14. Some unilateral injection groups showed minor non-significant threshold fluctuations POD5-7, with full recovery POD14-21.

**Figure 7:**
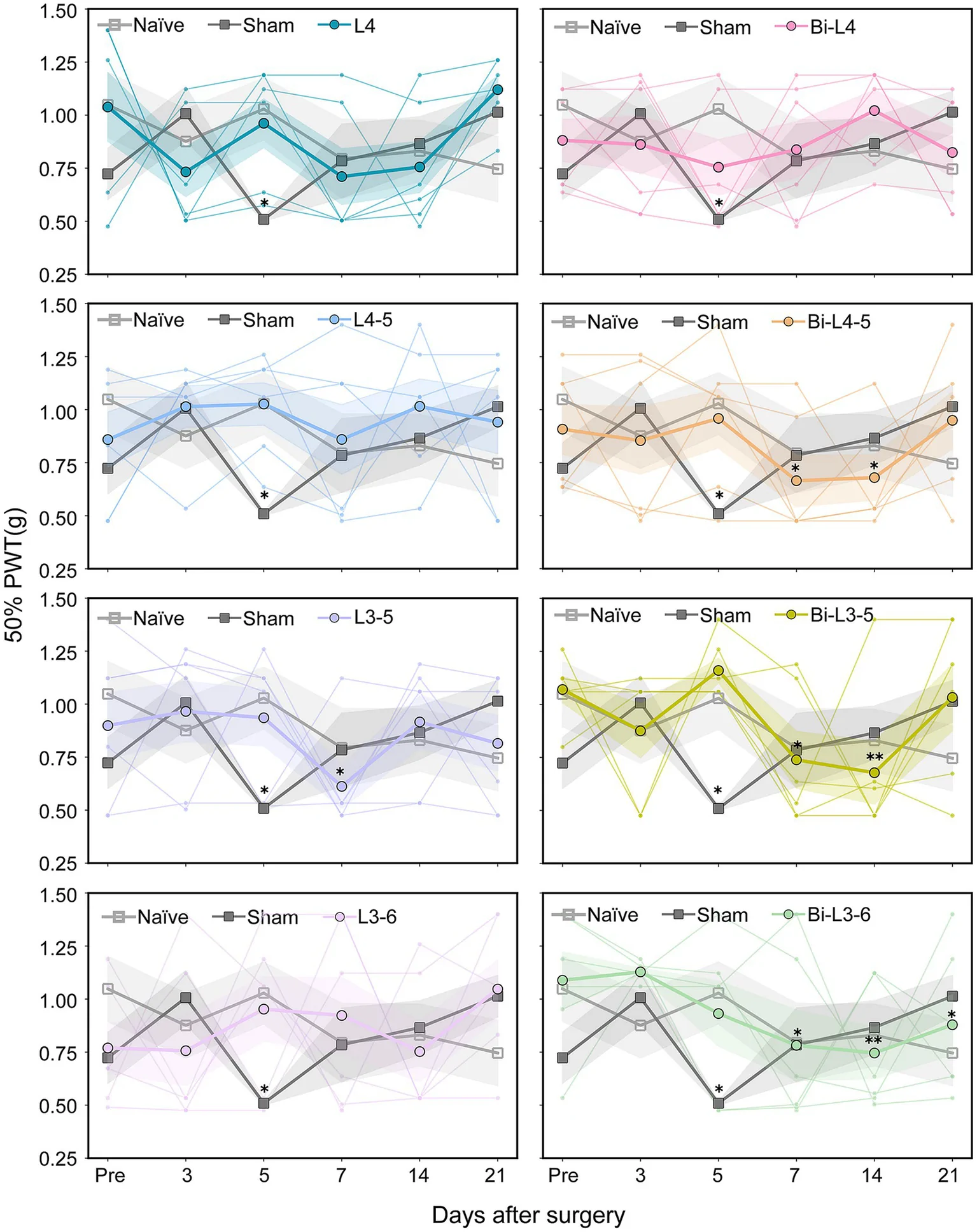
Temporal alterations in 50% paw withdrawal threshold (PWT, g). The allocation of the groups, the sample size (*n* = 6 per group) and the time of testing was chosen in agreement with that used in Figure 6. All statistical comparisons were performed within each group, comparing each postoperative time point against its own preoperative baseline (Pre). A two-way repeated-measures mixed-design ANOVA with Benjamini–Hochberg FDR-corrected post-hoc pairwise tests was applied for statistical comparisons. *p < 0.05; **p < 0.01; ***p < 0.001. Sham mice displayed mild transient mechanical hypersensitivity at postoperative days 3 to 7, which completely normalized by post-operative day 14. Unilateral injection groups showed minor non-significant fluctuations, with most groups achieving full recovery by POD 21. The bilateral DRG injection resulted in reversible segment-dependent mechanical allodynia that peaked between POD 7 and POD 14 and largely.

Bilateral injection triggered reversible mechanical hypersensitivity proportional to manipulated segment count: Bilateral L4 single segment: no significant threshold decline [max POD7 t (5)=1.209,*p* = 0.281]; Bilateral L4-5: significant drop starting POD7 [max 30.7% reduction, t (5)=3.347,*p* = 0.020], fully resolved POD21; Bilateral L3-5: hypersensitivity peaked POD14 [41.2% reduction, t (5)=4.816, *p* = 0.004], normalized POD21; Bilateral L3-L6: most prolonged sensitization [41.0% reduction at POD14, t (5)=4.702, *p* = 0.005], substantial recovery at endpoint.

A cross-sectional one-way ANOVA at POD7 confirmed significantly lower thresholds in bilateral multi-segment groups compared with sham and naïve controls [*F*(9,50) = 8.143, *p* < 0.001]. By POD21, naïve, sham, and unilateral groups had recovered completely. In the bilateral groups, a mild residual hypersensitivity was still detectable at POD21, although it was markedly attenuated compared with the peak values. Due to the absence of a vehicle-injected control group and histological examination, we cannot definitively attribute this residual effect to viral transduction versus persistent surgical trauma; therefore, we describe this observation as a partial recovery rather than a complete return to baseline. The overall recovery trend, however, supports the procedural safety of this injection approach.

### Transient motor impairment followed by robust Rotarod learning effect

Two-way mixed ANOVA revealed significant main effect of time [*F*(5,250) = 47.36, *p* < 0.001], non-significant group main effect [*F*(9,50)=1.07,*p* = 0.395], and group × time interaction [*F*(45,250) = 3.64, *p* < 0.001] (Figure 8). Although transient motor deficits were still observable at POD3, these deficits gradually diminished. Intergroup differences completely resolved by POD14, and all groups achieved comparable performance at POD14 and POD21. Post-hoc comparisons demonstrated progressive improvement in latency starting at POD5, with sustained elevation above preoperative baseline throughout POD5–14, consistent with repeated rotarod test learning effects. All transient motor coordination deficits resolved by POD 5.

**Figure 8:**
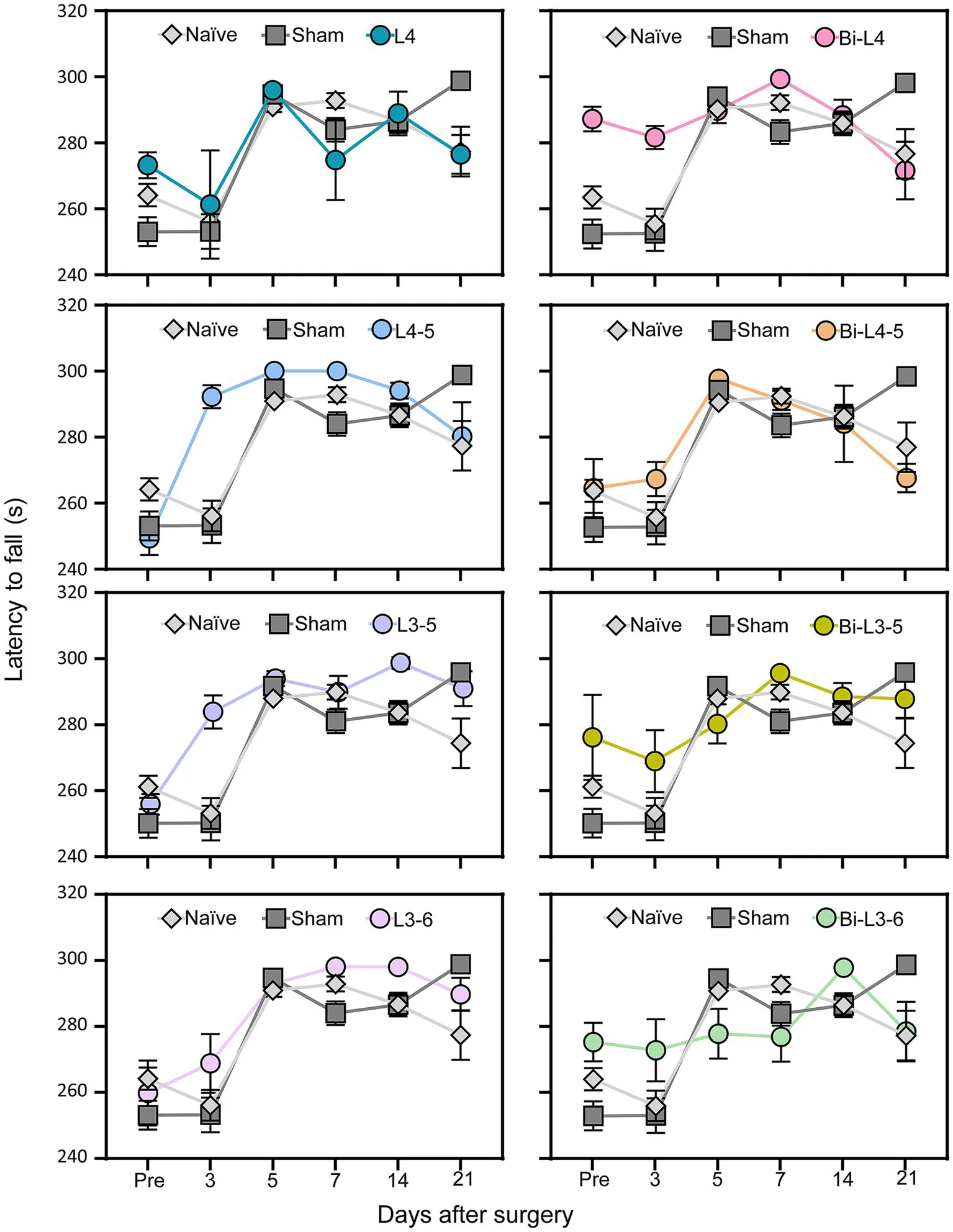
Serial rotarod fall latency (s) reflecting post-surgical motor coordination and balance function. As depicted in Figure 6, the grouping, sample size (*n* = 6 per group), and detection time points were the same as those in Figure 6. The symbols represent the group mean fall latency, and the error bars show the SEM. At POD 3, there were temporary minor motor deficits found in all surgical groups. Further, intergroup functional differences completely disappeared at POD 14 and at POD 21. Additionally, all groups had similar progressive latency improvement due to repeated test learning from POD 5. None of the groups had persistent motor deficits. Using a two-way repeated-measures mixed-design ANOVA analysis, scientists found a significant main effect of time and group × time interaction (*p* < 0.05) but a non-significant main effect of group (*p* = 0.360).

### Transient locomotor suppression with functional recovery at POD 21

Two-way mixed ANOVA detected significant main effects and interactions for mean open-field velocity: group [*F*(9,50)=2.27, *p* = 0.034, partial η^2^ = 0.33], time [*F*(5,250) = 10.62, *p* < 0.001, partial η^2^ = 0.21], group × time interaction [*F*(45,250) = 1.68, *p* = 0.007, partial η^2^ = 0.27]. One-way repeated-measures ANOVA confirmed significant time effect for peak velocity [*F*(5,295) = 9.132, *p* < 0.001]. Supplementary velocity distribution analysis revealed significant time-dependent shifts in immobility ratio, average speed, low/high-speed proportion (all *p* < 0.05, by repeated-measures ANOVA) (Figures 9, 10), indicating transient locomotor suppression. Global top velocity universally decreased POD3 and POD5 before gradual recovery. By POD21, all groups recovered baseline locomotor metrics entirely, even the most extensive bilateral injection cohort, confirming no permanent motor dysfunction induced by this surgical workflow.

**Figure 9:**
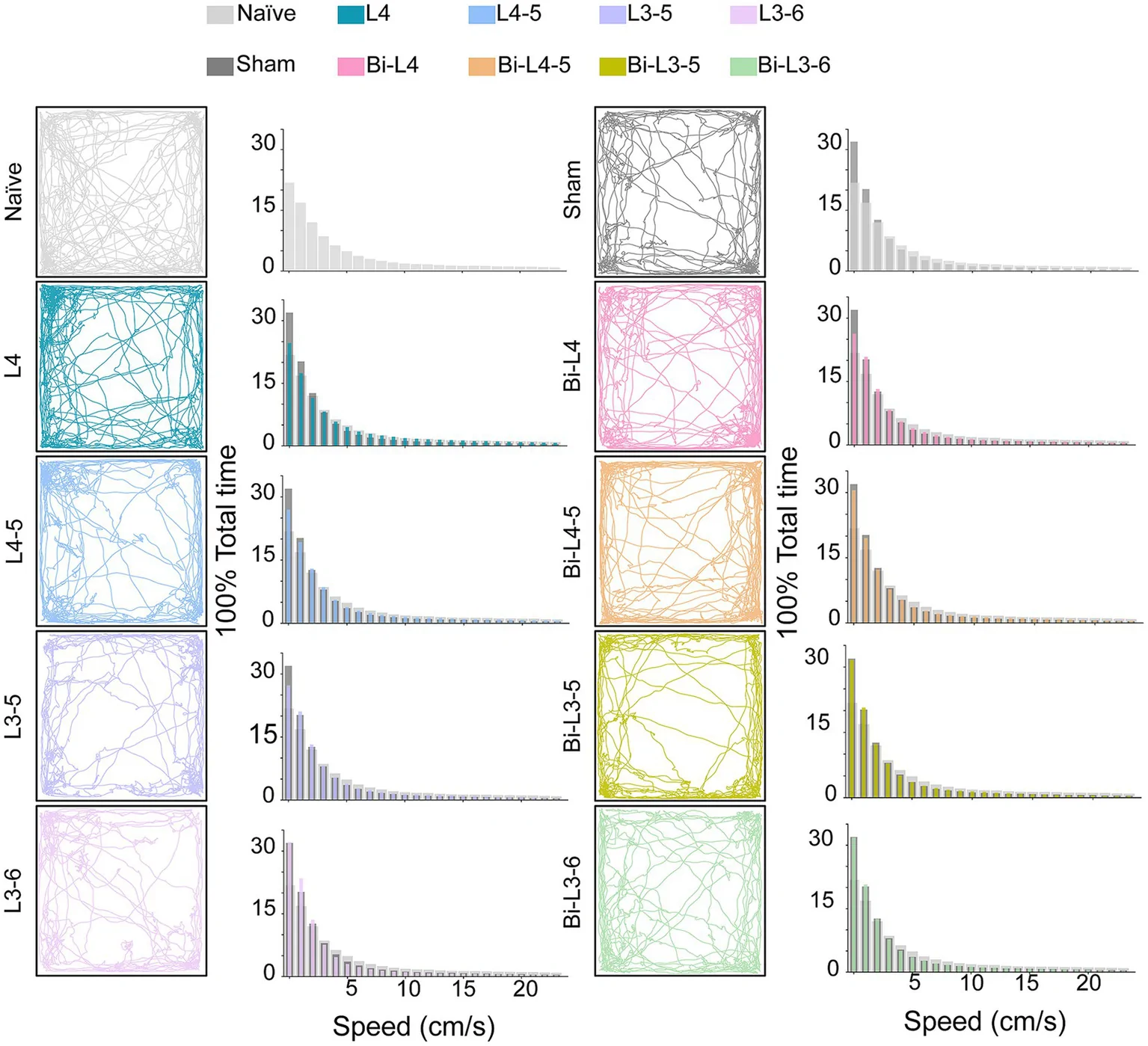
Spontaneous locomotor activity from open field testing across all experimental groups. Left: representative trajectories of an implanted and a sham-operated mouse during a 10-min open field test 5 days after surgery. Right: Speed distribution of implanted and sham-operated mice (*n* = 6 mice per group).

**Figure 10:**
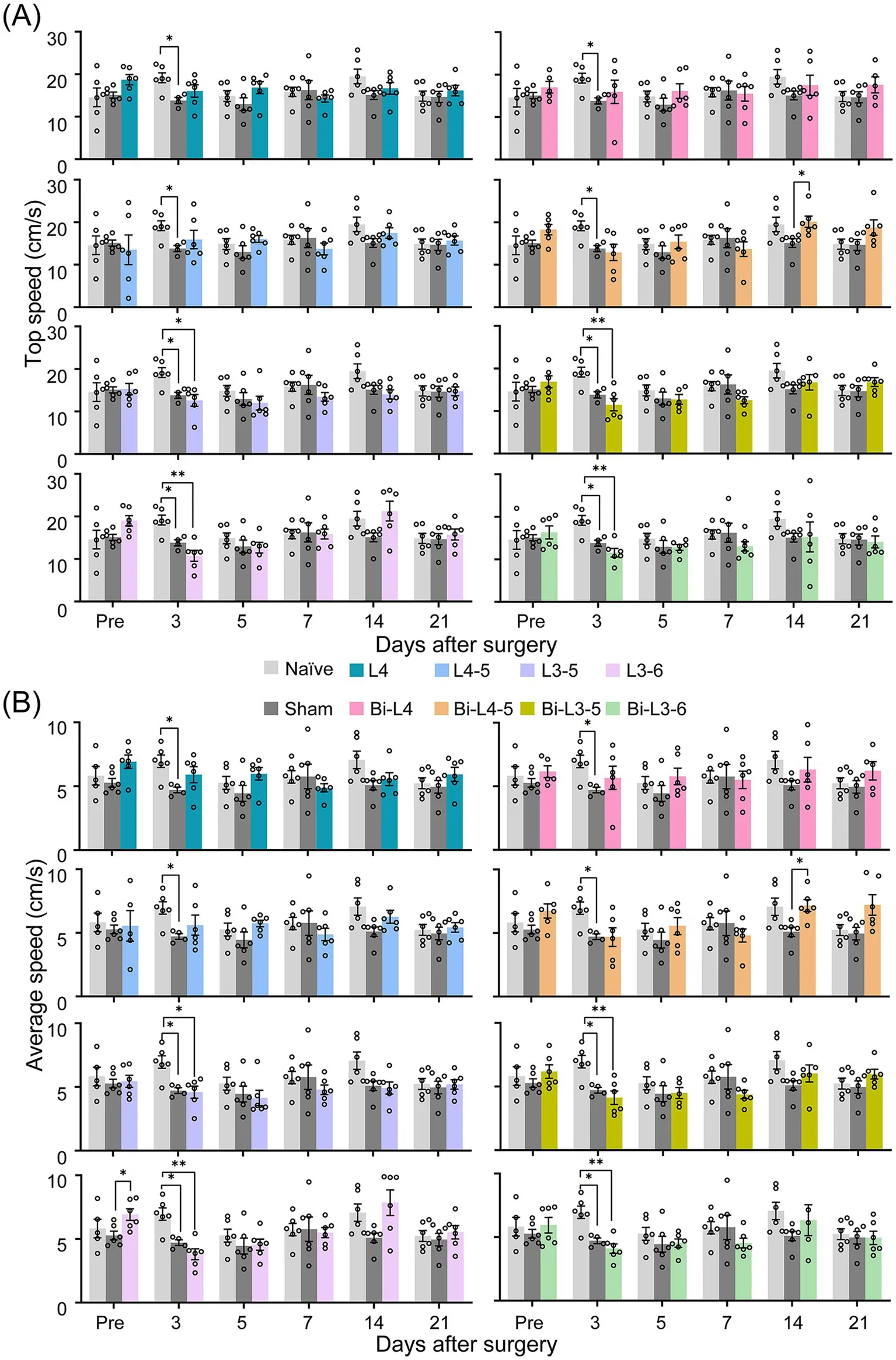
Changes in open-field locomotor speed over time following DRG injection. The serial top movement speed (cm/s) and serial average movement speed (cm/s) at Pre, POD 3, 5, 7, 14, 21 were reduced at POD 3–5 in all surgical groups, with slower hind limb recovery in bilateral multi-segment cohorts. All surgical groups restored to baseline levels by POD 21. Statistics: two-way repeated measures mixed-design ANOVA, group, time and group × time interaction effects were significant (*p* < 0.05). In contrast to the Naïve and Sham controls, injection groups were reviewed at each time point with FDR-corrected post-hoc tests. *p < 0.05, **p < 0.01, ***p < 0.001.

## Discussion

We established a surgical procedure that uses bony landmarks of the facet joints to guide restricted bone drilling, allowing simultaneous multi-target injection into the DRGs of lumbar segments L3-L6 in mice. To ensure reliable segment identification, the V-shaped white line of the spinous process was used as a reference, and vertebrae were counted from the caudal to cranial direction, starting from the permanently marked L6 vertebra—this approach was applied both in the initial surgery and in the second exposure at 21 days post-operation. YFP axon tracing was performed to map the innervation territories of the segmental DRGs, distinguishing the L3-L5 fibers that contribute to the sciatic nerve from the L6 fibers that supply pelvic visceral and pudendal afferents. The facet joints were used as additional bony landmarks to define the drilling points at the base of the inferior articular processes of the superior vertebrae. A custom temporary spinal stabilizer was employed during the procedure to maintain stability. Postoperative recovery was monitored through continuous behavioral assessments up to 21 days after surgery. Finally, in vivo calcium imaging was performed to confirm functional viral transduction.

### Vertebral landmark identification: reproducible intraoperative reference

Several previous reports have noted that many rodent DRG studies have misidentified vertebral segments (35). In this study a non-absorbable suture was used to mark the L6 spinous process to overcome this limitation. All anatomical samples were later confirmed through the T13 rib counting gold-standard method, proving the correctness of our landmark-based localization (30, 35). Studies on minimally invasive spine procedures presently highlight the need for clear and reliable vertebral landmarks to reduce target-localization errors (33, 39, 40). This landmark-based localization protocol is compatible with classic surgical approaches to the mouse dorsal root ganglia and provides a stable and reliable anatomical platform for experiments targeting the lumbar DRGs.

Notably, we did not strictly distinguish a true L6 vertebra from a sacralized caudal element during anatomical processing, given the well-documented prevalence of lumbosacral transitional vertebral anomalies in mice, including lumbar sacralization and sacral lumbarization (35). In our counting protocol, referencing the 13th rib consistently identified the six vertebral segments caudal to this rib, irrespective of whether the terminal segment represented a genuine L6 or a fused lumbosacral element. Importantly, previous studies have established that, despite such bony segmentation variability, the functional segmental innervation of the sciatic nerve remains invariant. Consequently, our landmark-based counting strategy effectively circumvents the ambiguity inherent in lumbosacral transitional anatomy and provides a reproducible, functionally relevant segmentation reference for DRG-targeted procedures.

### Anatomical rationale for complete L3-L6 lumbar coverage

In this study, YFP transgenic mice were used to trace segmental afferent projections. Our anatomical tracing provided clear evidence that, in C57 and C57-derived strains, sciatic nerve afferents originate exclusively from the L3–L5 DRGs, with no contribution from L1 or L2. In contrast, L6 afferent fibers travel along a separate pathway, do not participate in the sciatic nerve, but together with small branches from L5, formed the pudendal nerve (35). Existing DRG injection protocols are restricted to targeting only one or two lumbar segments, failing to achieve full coverage of sciatic nerve-related segments and hindering parallel controlled investigations into somatic-visceral sensory circuit interactions (13–18, 26, 30, 31). Our method achieves complete coverage of the L3-L6 segments, thereby facilitating investigations into multi-segmental afferent fiber convergence and mechanisms of referred pain between distinct sensory zones (13–18, 41). Notably, as peripheral nerve trunks contain both sensory and motor fiber populations, the present approach cannot precisely discriminate sensory afferents from motor efferents; for instance, it remains unclear whether sparse L3-derived fibers projecting toward L1-L2 nerve trunks and the small L5-derived branches within the pudendal nerve are sensory or motor branches. Further anterograde and retrograde segmental tracing will be required to resolve this issue in future work.

Furthermore, in general, the DRGs from L3 to L6 were not centrally aligned with their corresponding facet joints. From L3 to L6, the rostral edge of each DRG progressively shifted rostrally relative to the corresponding facet joint. This shift was most pronounced at L6, where even the caudal edge of the DRG lay rostral to the rostral edge of the L6/S1 facet joint. Of note, the bone drilling protocol follows the facet joints anatomy and is modular and versatile, not limited to fixed segment combinations. Depending on their experimental goals, selective targeting of one or other or multiple target points within the L3-L6 lumbar segments can be chosen by the researchers which will have their selective applicability for different experiment requirements and pain research models.

### Landmark-based drilling limits bone removal and spares the facet joint

Taking the base of the articular process as a drilling target, we established a surgical approach to the DRG without damage to the facet joint capsule and/or articular cartilage surface. The lumbar facet joints are physiological synovial structures that regulate the directional movement of the spine, while maintaining stability of the vertebrae (32, 33). The conventional DRG injections require significant laminectomy or resection of the posterior facet joint to visualize the ganglion, which leads to a considerable amount of bone removal and weakening of the facet joint structure. Excessive resection of bone can result in postoperative spinal biomechanical instability and abnormal vertebral motion in experimental animals, as well as a chronic local inflammatory response that causes chronic pain (32, 34, 39, 42–44).

According to the core principles of stereotactic neurosurgery originally devised for intracerebral viral delivery, the drilling pathway in this study relies on bony landmarks for localization and minimally invasive removal of bone (45, 46). This drilling method uses anatomical landmarks for positioning while preserving the facet joints. Unlike earlier techniques that required partial facet joint resection for access to the DRG (30), this drilling strategy is intended to reduce the risk of iatrogenic instability by preserving the facet articular surface. By day 21 post surgery, the animals had apparently returned to normal behavioural function, and the absence of tissue adhesions seen at final necropsy supports the safety of this procedure. Although, our protocol does not reduce the extent of soft tissue dissection over the other injection protocols; however, its main advantage is the restriction of bone resection, which avoids the main drawback related to excessive resection of spinal bones in conventional surgeries.

### Stimulus-evoked calcium imaging provides functional evidence of reporter expression

Using two-photon microscopy, we observed spontaneous calcium transients in the dorsal root ganglion neurons of anesthetized GCaMP6s transgenic mice (47). In contrast, DRG neurons expressing jGCaMP8s under the same anesthetic conditions exhibited almost no spontaneous calcium activity when examined with the Axio Zoom.V16 macro-zoom fluorescence system. This initially suggested to us that viral transduction had failed. These inconsistencies might be due to differences in anesthetic depth, on one hand, and differences in the kinetic properties of GCaMP6s and jGCaMP8s probes (48–52), on the other hand. Detection sensitivity of two-photon and regular single-photon microscopy may also differ.

The fluorescence imaging was performed at baseline static fluorescence stage whereby wild-type mice and rAAV injected mice exhibited granular spontaneous fluorescence that did not allow us to evaluate the transgenic expression. Inflation of air currents overcame this limitation whereby physiological neuronal calcium transients were elicited as clear functional evidence. Under identical stimulation and imaging conditions, wild-type negative control mice exhibited no calcium signalling, showing that any baseline granular fluorescence likely came from non-specific autofluorescence rather than functional jGCaMP8s. The results suggest that the use of static fluorescence imaging is not sufficient to confirm successful injection of DRG with virus; thus, stimulation-evoked functional calcium recordings represent an important validation step in the workflow of the experiment (48, 49).

### Postoperative recovery confirms low surgical morbidity

Through this approach, the identification of prognostic features associated with few postoperative complications and a predictable outcome is possible. The absorbable sutures detached spontaneously on days 5–7 post-surgery as the hair follicles reformed which made it unnecessary for removal. By day 21, the skin morphology had returned to the baseline condition. The final analysis showed that both the spinal cord and DRG surfaces are smooth and there are no adhesions. There are also no obvious inflammatory vascular proliferations. The recovery trajectory observed by day 21 was generally consistent with that previously reported for rodent invasive spinal procedures (26, 31, 47).

### Comparative analysis against conventional DRG injection protocols

It should be noted that the present study does not include side-by-side in-vivo experimental benchmarking against conventional DRG injection protocols. Relative advantages of our technique are partially inferred from well-documented drawbacks of existing approaches reported in previous literature, rather than derived from direct parallel animal comparisons. Conventional open DRG microinjection methods typically require substantial bone resection or facet joint removal, which predispose animals to spinal instability, severe postoperative tissue adhesion, and limited feasibility for multi-segment targeting. Our longitudinal behavioral monitoring and terminal gross anatomical observations demonstrate minimized bone removal, absence of obvious tissue adhesion, and reliable multi-segment access to L3-L6 DRGs, suggesting that our workflow mitigates these documented surgical shortcomings. Future dedicated head-to-head comparative studies will be valuable to quantitatively benchmark key practical parameters including surgical duration, the magnitude of bone resection, injection success rate, and the severity of post-operative adhesion across different surgical strategies.

Yuan et al. (26) developed an open lumbar DRG injection technique that can reduce the extent of total laminectomy injury. However, this approach requires resection of substantial portion of the articular process, which may compromise spinal stability to some degree. The procedure is primarily described for contiguous lumbar segments and may be limited by scar adhesion formation if reoperation is attempted. Additionally, the original description does not specify whether the animals were immobilized during the viral injection, which may increase the risk of DRG injury due to respiratory or cardiac motion. While the authors suggested that the method could be extended to other levels, the experimental validation in that study was limited to the L4 DRG. O’Donnell et al. (30) described a partial laminectomy method to enlarge the intervertebral foramen for DRG access, and they demonstrated successful injection into L3 and L4 DRGs with the potential for adaptation to other levels. However, as with the Yuan study, the extension to cervical or thoracic levels was proposed but not experimentally validated in their report. In contrast to the above methods, our approach incorporates a custom spinal stabilizer to minimize motion-related DRG injury during injection, and achieves simultaneous, one-step coverage of L3-L6 DRGs while largely preserving the spinal osseous structure and facet joint integrity. This multi-segmental capability enables concurrent targeting of both somatic nociceptive (L3-L5) and pelvic visceral (L6) afferent pathways, which may facilitate studies on somato-visceral sensory integration that are not readily addressed by single-segment injection techniques.

### Study limitations and future experimental directions

This study has several limitations. Post-operative follow-up was limited to day 21, and the protocol was only validated in C57BL/6 J mice without testing in pain models. We did not systematically profile AAV serotype-dependent transduction efficiency and dose-related neurotoxicity (23, 24, 28). Although our drilling minimizes bone resection and preserves gross facet-joint structures, safety evaluation depends on behavioral and gross anatomical observations, lacking direct quantitative spinal biomechanical evidence from micro-CT, cartilage histology or ex-vivo biomechanical assays. Functional validation relies on macroscopic wide-field calcium imaging, precluding single-neuron-resolved calcium quantification. We also lack segment-wise immunohistochemical quantification of transduced neurons and systematic multi-tissue assessment of viral leakage and off-target transduction. While relevant vector properties have been reported elsewhere, future dedicated experiments are needed for full structural-functional characterization, and end-users should perform customized profiling tailored to their experimental goals.

## Conclusion

In summary, we present a surgical protocol that utilizes the articular process bony landmarks to enable concomitant and focused targeting of multiple DRG segments in mice via localized removal of bone. The procedure fully covers the L3-L6 segments, thereby allowing for mechanistic studies of two different primary sensory circuits. This approach can produce consistent results for most target levels, entails minimal bone damage, and yields largely transient postoperative functional deficits that resolve in most animals. Importantly, a subset of bilateral L3-L6 multi-segment-injected mice retain residual hypersensitivity at POD 21 and require pre-experimental screening. The custom spinal stabilizer provides intraoperative immobilization, and the protocol allows flexible selection of target segments within L3-L6. With these caveats, the platform offers a practical tool for studying the integration of multi-segmental somatosensory and viscerosensory inputs, as well as for preclinical and translational applications of DRG-targeted gene therapy in chronic pain disorders.

## Data Availability

The original contributions presented in the study are included in the article/Supplementary material, further inquiries can be directed to the corresponding author/s.
