# Supplementary material for: A facet joint landmark-guided approach for multi-segmental DRG injection with limited bone removal in mice
Source: Front Neurol. 2026 Oct 15;17:1956927. doi: 10.3389/fneur.2026.1956927 (PMC13716812; doi:10.3389/fneur.2026.1956927)
Supplement: Supplementary file 1 [file Data_Sheet_1.zip › Supplementary File/Mouse Spinal Stabilizer-2D-pdf/Mouse Spinal Stabilizer - Upright Post.pdf]

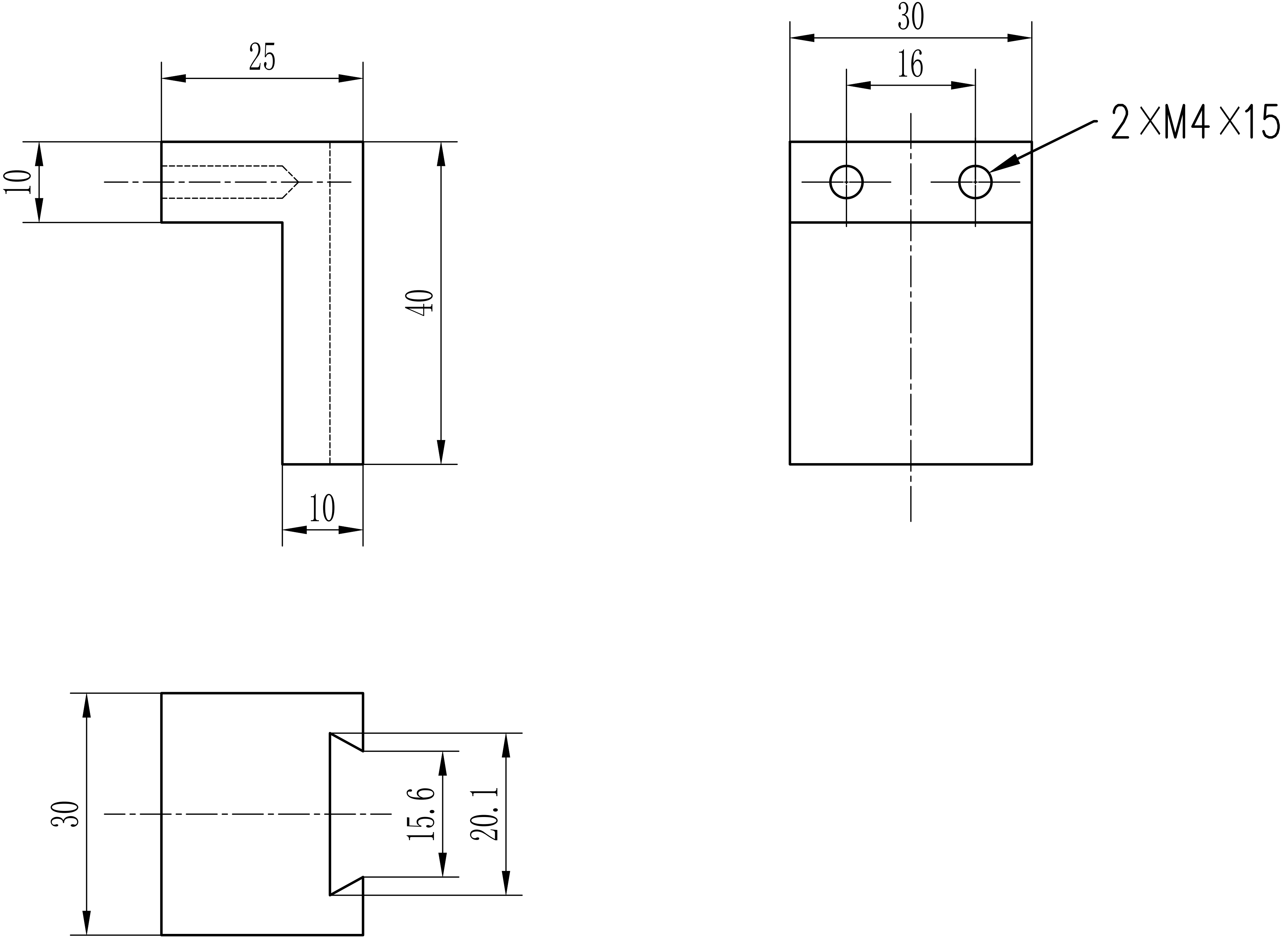
25
10
40
10
30
16
2×M4×15
30
15.6
20.1
