# Supplementary material for: A facet joint landmark-guided approach for multi-segmental DRG injection with limited bone removal in mice
Source: Front Neurol. 2026 Oct 15;17:1956927. doi: 10.3389/fneur.2026.1956927 (PMC13716812; doi:10.3389/fneur.2026.1956927)
Supplement: Supplementary file 1 [file Data_Sheet_1.zip › Supplementary File/Mouse Spinal Stabilizer-2D-pdf/Supplementary Notes.pdf]

Supplementary Notes: Material and Fabrication Details

All components of the custom mouse spinal stabilizer are manufactured from **6061-T6 aluminum alloy.** This material was selected for its high stiffness-to-weight ratio, good machinability, corrosion resistance, and ease of surface cleaning for laboratory animal experiments.

General machining requirements

1. All dimension units shown on drawings are **millimetres**.
2. General dimensional tolerance: ±0.1 mm for unmachined nominal dimensions; ±0.05 mm for fit-related dimensions between sliding interfaces of the Sliding Block and mating parts.
3. Sharp edges and burrs on all components shall be fully deburred to avoid potential injury to experimental animals and operators.
4. Surface finish: machine-finished surface without additional coating; parts can be disinfected with 75% ethanol before animal experiments.

Hole-specific requirements

1. **Countersink through holes (CSK through hole, M4):** Conical countersink for M4 countersunk-head screws; holes are fully through without internal threads, allowing screws to pass through.
2. **Countersunk tapped holes (CSK tapped hole, M4 / M2.5):** Conical countersink combined with internally tapped threads, for fastening with countersunk-head screws.
3. Threaded holes (e.g. 2 × M4 × 15 on Upright Post): Standard metric internal thread, thread depth follows drawing annotation.

Components overview

1. **Base Plate:** The foundation platform, machined with multiple M4 countersink through-holes for assembly and position adjustment of upright posts.
2. **Upright Post:** L-shaped supporting bracket, fixed onto the base plate; contains threaded holes for mounting the sliding block assembly.
3. **Sliding Block:** Sliding adjustable component with M2.5 tapped hole and M4 countersunk tapped hole, enabling positional adjustment of clamping jaws.
4. **Clamping Jaw:** Thin-wall clamping part for spinal fixation of mice; precise small-dimension machining is required for its clamping end.

The provided editable CAD files allow researchers to modify part dimensions if adaptation for experimental animals of different body sizes is required.
